# Sodium selenite inhibits deoxynivalenol-induced injury in GPX1-knockdown porcine splenic lymphocytes in culture

**DOI:** 10.1038/s41598-018-36149-x

**Published:** 2018-12-05

**Authors:** Zhihua Ren, Yu Fan, Zhuo Zhang, Chaoxi Chen, Changhao Chen, Xuemei Wang, Junliang Deng, Guangneng Peng, Yanchun Hu, Suizhong Cao, Shumin Yu, Xiaoping Ma, Liuhong Shen, Zhijun Zhong, Ziyao Zhou, Zhiwen Xu, Zhicai Zuo

**Affiliations:** 1 College of Veterinary Medicine, Sichuan Agricultural Uniiversity, Sichuan Province Key Laboratory of Animal Disease and Human Health, Key Laboratory of Environmental Hazard and Human Health of Sichuan Province, Chengdu, 611130 China; 2 Collge of Life Since and Technology, Southwest Minzu University, Chengdu, 611130 China

## Abstract

Deoxynivalenol (DON) is a cytotoxic mycotoxin that can cause cell damages. The main effect is to inhibit protein synthesis. Oxidative stress is one of the effects of DON. Selenium (Se) can ameliorate the cell damage caused by DON-induced oxidative stress, but it is unclear whether through selenoprotein glutathione peroxidase 1 (GPX1). We established GPX1-knockdown porcine spleen lymphocytes, and treated them with DON and Se. Untransfected porcine splenic lymphocytes (group P) and transfected cells (group M, GPX1 knockdown) were treated with or without DON (0.824, 0.412, 0.206, or 0.103 μg/mL, group D1-4), Se (Na_2_SeO_3_, 2 μM, group Se), or both (group SD1–4) for 6, 12, or 24 h. The cells were collected and the activities of SOD and CAT, levels of GSH, H_2_O_2_, malonaldehyde (MDA), total antioxidant capacity (T-AOC), and the inhibition of free hydroxyl radicals were determined. Levels of ROS were measured at 24 h. Compared with group P, the antioxidant capacity of group M was reduced. DON caused greater oxidative damage to the GPX1-knockdown porcine splenic lymphocytes than to the normal control cells. When Na_2_SeO_3_ was combined with DON, it reduced the damage in the GPX1-knockdown porcine splenic lymphocytes, but less effectively than in the normal porcine splenic lymphocytes.

## Introduction

Deoxynivalenol (DON) is a stable trichothecene mycotoxin^1^, so it is difficult to destroy or eliminate during conventional food storage or processing. Therefore, it readily causes zoonoses^2^. Different species of animals display different tolerance for DON, and pigs are highly sensitive to it^3^. DON not only reduces the utilization rate of animal feed, but also reduces the growth performance and reproductive performance of animals and destroys their immune systems^4^. The spleen is the main target when DON affects the immune system. DON affects cell signalling^5^, interferes with and damages ribosomes^2^, inhibits the synthesis of proteins and nucleic acids^5,6^, and promotes cell apoptosis^7,8^. Oxidative stress is an important mechanism of DON-mediated cytotoxicity and apoptosis^9^. The main mechanism by which DON induces oxidative stress is by the accumulation of high levels of reactive oxygen species (ROS) in the cell, destroying the cellular oxidation–antioxidant balance^10^. ROS induce lipid peroxidation in the lipid membrane, damaging its phospholipids and lipoproteins, and causes DNA damage in a chain reaction^8,11^.

Selenium (Se) is a necessary trace element for animals, including humans^12^, and is especially required by the immune system^13^. Selenium has many biological functions, the most important of which is in anti-oxidation. Selenium is the most important component of the glutathione peroxidase (GPX) active centre, selenocysteine, and participates in important processes by inhibiting lipid peroxidation, catalysing the reduction by glutathione (GSH) of toxic peroxides in the body, removing excessive free radicals, and protecting the mechanisms and functions of the cell membrane. A large number of studies have shown that the addition of the proper amount of Se enhances the antioxidant capacity of the body or cell and increases the expression of GPX1^14^. GPX1 also has some preventive effects on the oxidative damage caused by mycotoxins^15–18^.

GPX1 was the first antioxidant enzyme shown to reduce H_2_O_2_ in red blood cells via GSH^19^. It is the most important antioxidant enzyme in the body and is widely expressed during major cell division. It can remove free radicals and peroxide from cells, and together with other antioxidant enzymes (catalase [CAT] and superoxide dismutase [SOD]), constitutes the endogenous antioxidant defence system^20,21^. An appropriate increase in GPX1 expression can enhance the antioxidant capacity of cells^22,23^.

Our laboratory has shown that Se can reduce the damage to porcine spleen lymphocytes caused by DON-induced oxidative stress^19^, and can prevent the concomitant changes in cytokines induced in porcine spleen lymphocytes^24^. However, it remains unclear whether it antagonizes DON toxicity through the selenoprotein GPX1. In this study, we established GPX1-knockdown porcine spleen lymphocytes and treated them sodium selenite (Na_2_SeO_3_) and DON, singly or combined, in a culture system. We then measured the intracellular antioxidant index and the ROS content of the GPX1-knockdown porcine spleen lymphocytes to determine the protective effects of sodium selenite on DON-induced oxidative damage in these cells and whether Se acts through the selenoprotein GPX1 in antagonizing the toxicity of DON.

## Results

### Transfection efficiency of GPX1-directed small interfering RNA (siRNA) in porcine spleen lymphocytes

**Figure 1:**
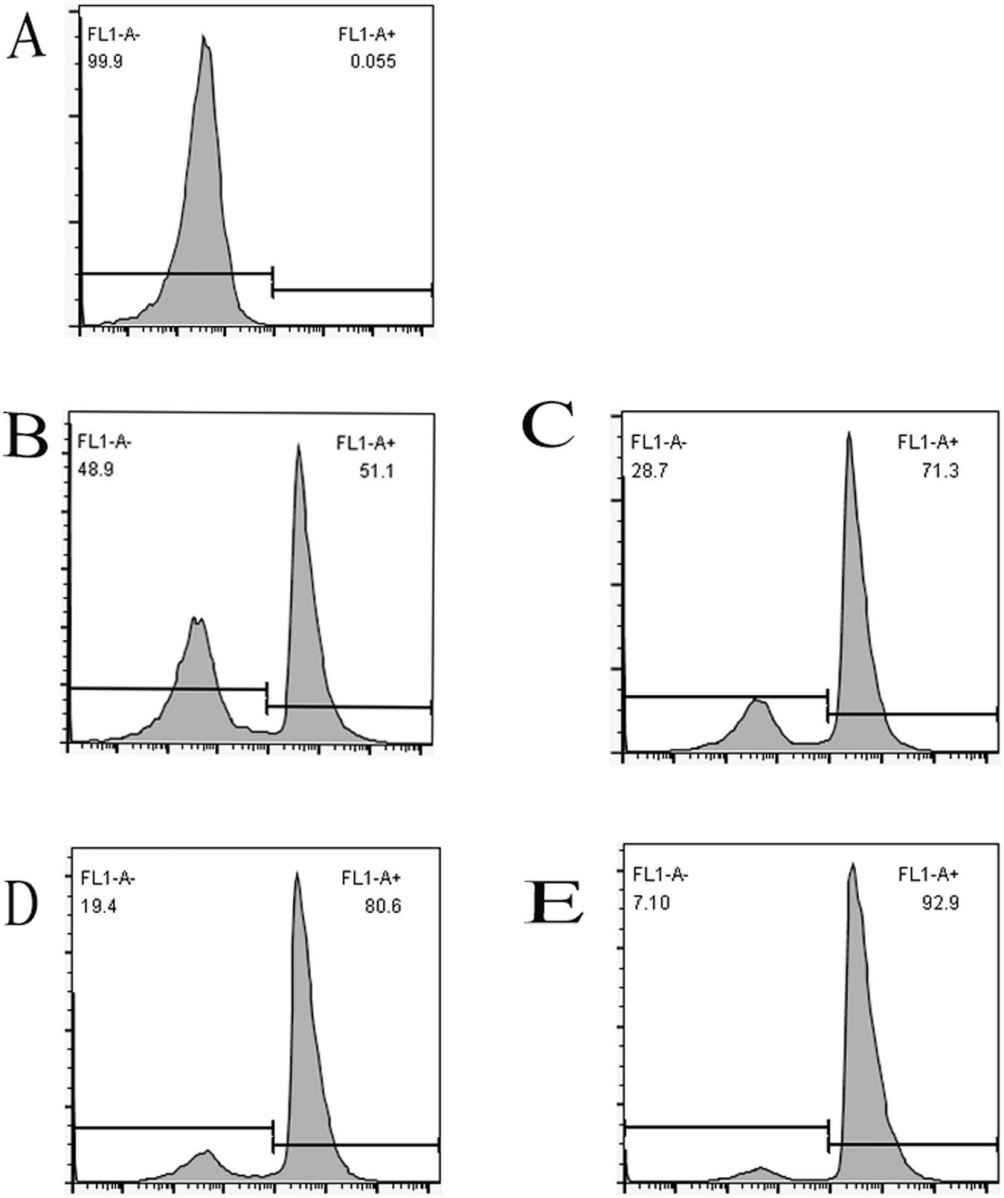
Transfection efficiency of GPX1-directed siRNA in porcine splenic lymphocytes. the blank control shown in (**A**), the transfection efficiency of combination was 51.1% + 0.8% shown in (**B**), the transfection efficiency of combination was 71.3% + 1.3% shown in (**C**), the transfection efficiency of combination was 80.6% + 1.7% shown in (**D**), and the transfection efficiency of combination was 92.9% + 2% shown in (**E**). Therefore, we selected combination E for the subsequent experiment.

The transfection efficiency of GPX1-directed siRNA in porcine spleen lymphocytes is shown in Fig. 1. Transfection efficiency of GPX1-directed siRNA in porcine splenic lymphocytes. the blank control shown in A. In B, C, D, E shown the different transfection efficiency of combination. Combination E has the best transfection effect, we selected combination E for the subsequent experiment.

### Expression of GPX1 mRNA after siRNA transfection

The relative expression of GPX1 mRNA after siRNA transfection is shown in Table 1. The expression of GPX1 in the group of cells treated with the control GPX1-directed siRNA was 28.4% of that in the control group, This suggests that there was nonspecific gene knockdown.

**Table 1:** Relative expression of GPX1 mRNA.

Groups	Control siRNA	Scrambled siRNA	Control
Relative expression (%)	28.4 ± 3.2	99.4 ± 4.8	100 ± 1.7

### Expression of GPX1 protein in porcine spleen lymphocytes after siRNA transfection

**Figure 2:**
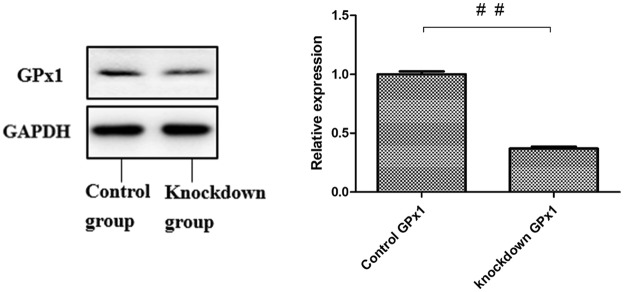
Relative expression of GPX1 and glyceraldehyde 3-phosphate dehydrogenase (GAPDH) proteins. Its show that there was no significant difference in the expression of GAPDH between the normal and knockdown groups. However, the expression of GPX1 differed significantly between the normal control and knockdown groups, and that GPX1-knockdown cells expressed only 36.9% of the GPX1 expressed by the normal group. Therefore, the knockdown efficiency was 63.1%. ^##^*P* < 0.01. Table 1. the expression of GPX1 in the group of cells treated with the control GPX1-directed siRNA was 28.4% of that in the control group, whereas the expression of GPX1 in the scrambled siRNA group was 99.4% of that in the control group. This suggests that there was no nonspecific gene silencing.

The expression of the GPX1 protein in porcine spleen lymphocytes after siRNA transfection is shown in Fig. 2. That GPX1-knockdown cells expressed only 36.9% of the GPX1 expressed by the normal group. Therefore, the knockdown efficiency was 63.1%.

### Antioxidant indices and ROS levels

The activities of SOD and CAT, the levels of GSH, hydrogen peroxide (H_2_O_2_), and malonaldehyde (MDA), the total antioxidant capacity (T-AOC), and the ability to inhibit free hydroxyl radicals are shown in Tables 2–8. The activities of SOD and CAT and the ability to inhibit free hydroxyl radicals were significantly lower in group M than in group P at most time points (*P* < 0.01); the level of GSH was significantly lower in group M than in group P at each time point, except at 6 h; and the levels of H_2_O_2_ and MDA were significantly higher in group M than in group P at each time point. Treatment with DON (0.824–0.103 μg/mL) alone reduced the activities of SOD and CAT, the levels of GSH and T-AOC, and the ability to inhibit free hydroxyl radicals significantly more strongly in group M than in the groups D1–4 at most time points; and the levels of H_2_O_2_ and MDA were significantly higher in the groups D1–4 than in group M. Treatment with Na_2_SeO_3_ (2 μmol/L) alone significantly increased the activities of SOD and CAT, T-AOC, and free hydroxyl radical inhibition relative to those in group M, except for GSH at 6 h. The levels of H_2_O_2_ and MDA were significantly lower in the groups D1–4 than in group M. When the lymphocytes were treated with both DON and Na_2_SeO_3_, the activities of SOD and CAT, the levels of GSH and T-AOC, and the inhibition of free hydroxyl radicals were significantly higher in group SD1–4 than in group D1–4 at most time points.

**Table 2:** Effects of DON and/or Na_2_SeO_3_ on the levels of H_2_O_2_ in GPX1-knockdown porcine splenic lymphocytes at 6, 12, and 24 h after treatment

H_2_O_2_ (mmol/gprot)				
time	pairing	content		*P*
6 h	P — M	10.978 ± 0.387	13.964 ± 0.730	0.004
	M — Se	13.964 ± 0.730	12.526 ± 0.422	0.015
	M — D1	13.964 ± 0.730	19.719 ± 0.970	0.001
	M — D2	13.964 ± 0.730	16.845 ± 0.269	0.008
	M — D3	13.964 ± 0.730	15.823 ± 0.415	0.009
	M — D4	13.964 ± 0.730	14.527 ± 0.632	0.010
	D1 — SD1	19.719 ± 0.970	19.341 ± 1.271	0.162
	D2 — SD2	16.845 ± 0.269	16.291 ± 0.732	0.174
	D3 — SD3	15.823 ± 0.415	15.037 ± 0.324	0.004
	D4 — SD4	14.527 ± 0.632	13.639 ± 0.283	0.048
12 h	P — M	12.206 ± 0.513	17.757 ± 0.815	0.001
	M — Se	17.757 ± 0.815	15.617 ± 0.689	0.001
	M — D1	17.757 ± 0.815	30.445 ± 1.663	0.001
	M — D2	17.757 ± 0.815	26.350 ± 1.513	0.002
	M — D3	17.757 ± 0.815	23.882 ± 1.438	0.003
	M — D4	17.757 ± 0.815	21.540 ± 1.633	0.015
	D1 — SD1	30.445 ± 1.663	29.654 ± 1.513	0.012
	D2 — SD2	26.350 ± 1.513	24.767 ± 1.168	0.015
	D3 — SD3	23.882 ± 1.438	22.273 ± 0.784	0.051
	D4 — SD4	21.540 ± 1.633	20.593 ± 0.816	0.182
24 h	P — M	19.759 ± 0.973	25.341 ± 1.110	<0.001
	M — Se	25.341 ± 1.110	22.547 ± 0.754	0.005
	M — D1	25.341 ± 1.110	35.623 ± 1.581	0.001
	M — D2	25.341 ± 1.110	32.710 ± 1.750	0.003
	M — D3	25.341 ± 1.110	28.664 ± 1.372	0.002
	M — D4	25.341 ± 1.110	27.633 ± 1.038	<0.001
	D1 — SD1	35.623 ± 1.438	33.779 ± 1.384	0.004
	D2 — SD2	32.710 ± 1.309	30.053 ± 1.268	0.011
	D3 — SD3	28.664 ± 1.025	24.524 ± 1.082	0.002
	D4 — SD4	27.633 ± 1.016	22.240 ± 1.120	<0.001

**Table 3:** Effects of DON and/or Na_2_SeO_3_ on the levels of MDA in GPX1-knockdown porcine splenic lymphocytes at 6, 12, and 24 h after treatment.

MDA (nmol/mg.prot)				
time	pairing	content		*P*
6 h	P — M	7.144 ± 0.027	7.584 ± 0.088	0.006
	M — Se	7.584 ± 0.088	7.353 ± 0.060	0.005
	M — D1	7.584 ± 0.088	10.276 ± 0.089	<0.001
	M — D2	7.584 ± 0.088	8.812 ± 0.042	<0.001
	M — D3	7.584 ± 0.088	8.114 ± 0.065	0.001
	M — D4	7.584 ± 0.088	7.487 ± 0.066	0.017
	D1 — SD1	10.276 ± 0.089	10.152 ± 0.132	0.037
	D2 — SD2	8.812 ± 0.042	8.586 ± 0.127	0.045
	D3 — SD3	8.114 ± 0.065	7.851 ± 0.072	<0.001
	D4 — SD4	14.527 ± 0.632	7.348 ± 0.073	0.001
12 h	P — M	7.573 ± 0.057	8.374 ± 0.098	0.001
	M — Se	8.374 ± 0.098	8.108 ± 0.137	0.007
	M — D1	8.374 ± 0.098	11.717 ± 0.195	<0.001
	M — D2	8.374 ± 0.098	10.398 ± 0.161	<0.001
	M — D3	8.374 ± 0.098	9.432 ± 0.086	<0.001
	M — D4	8.374 ± 0.098	8.521 ± 0.074	0.009
	D1 — SD1	11.717 ± 0.195	11.489 ± 0.270	0.034
	D2 — SD2	10.398 ± 0.161	10.111 ± 0.190	0.003
	D3 — SD3	9.432 ± 0.086	9.074 ± 0.198	0.031
	D4 — SD4	8.521 ± 0.074	8.105 ± 0.186	0.023
24 h	P — M	8.192 ± 0.109	9.207 ± 0.724	0.104
	M — Se	9.207 ± 0.724	8.940 ± 0.082	0.546
	M — D1	9.207 ± 0.724	12.872 ± 0.260	0.005
	M — D2	9.207 ± 0.724	11.878 ± 0.245	0.011
	M — D3	9.207 ± 0.724	11.011 ± 0.046	0.044
	M — D4	9.207 ± 0.724	10.201 ± 0.160	0.093
	D1 — SD1	12.872 ± 0.260	12.435 ± 0.235	0.001
	D2 — SD2	11.878 ± 0.245	10.923 ± 0.274	<0.001
	D3 — SD3	11.011 ± 0.046	10.327 ± 0.237	0.025
	D4 — SD4	10.201 ± 0.160	9.439 ± 0.108	0.002

**Table 4:** Effects of DON and/or Na_2_SeO_3_ on SOD activity in GPX1-knockdown porcine splenic lymphocytes at 6, 12, and 24 h after treatment.

SOD activities(U/mg.prot)				
time	pairing	activities		*P*
6 h	P — M	52.592 ± 1.104	47.394 ± 0.872	0.001
	M — Se	47.394 ± 0.872	48.646 ± 0.653	0.010
	M — D1	47.394 ± 0.872	31.763 ± 0.340	<0.001
	M — D2	47.394 ± 0.872	37.786 ± 0.272	0.001
	M — D3	47.394 ± 0.872	44.982 ± 0.590	0.005
	M — D4	47.394 ± 0.872	47.700 ± 0.816	0.011
	D1 — SD1	31.763 ± 0.340	31.923 ± 0.399	0.042
	D2 — SD2	37.786 ± 0.272	38.516 ± 0.657	0.082
	D3 — SD3	44.982 ± 0.590	46.158 ± 0.565	<0.001
	D4 — SD4	47.700 ± 0.816	49.241 ± 0.803	<0.001
12 h	P — M	46.305 ± 0.566	43.505 ± 0.502	<0.001
	M — Se	43.505 ± 0.502	45.169 ± 0.697	0.005
	M — D1	43.505 ± 0.502	25.931 ± 0.364	<0.001
	M — D2	43.505 ± 0.502	33.502 ± 0.307	<0.001
	M — D3	43.505 ± 0.502	37.588 ± 0.596	<0.001
	M — D4	43.505 ± 0.502	40.446 ± 0.595	<0.001
	D1 — SD1	25.931 ± 0.364	26.253 ± 0.435	0.016
	D2 — SD2	33.502 ± 0.307	34.266 ± 0.537	0.029
	D3 — SD3	37.588 ± 0.596	38.659 ± 0.387	0.012
	D4 — SD4	40.446 ± 0.595	43.253 ± 0.503	<0.001
24 h	P — M	39.245 ± 0.427	32.629 ± 0.546	<0.001
	M — Se	32.629 ± 0.546	33.907 ± 0.531	<0.001
	M — D1	32.629 ± 0.546	16.264 ± 0.229	<0.001
	M — D2	32.629 ± 0.546	18.606 ± 0.140	<0.001
	M — D3	32.629 ± 0.546	21.386 ± 0.347	<0.001
	M — D4	32.629 ± 0.546	25.740 ± 0.231	0.001
	D1 — SD1	16.264 ± 0.229	16.870 ± 0.248	<0.001
	D2 — SD2	18.606 ± 0.140	19.399 ± 0.368	0.026
	D3 — SD3	21.386 ± 0.347	25.551 ± 0.500	<0.001
	D4 — SD4	25.740 ± 0.231	30.201 ± 0.334	<0.001

**Table 5:** Effects of DON and/or Na_2_SeO_3_ on the CAT activity in GPX1-knockdown porcine splenic lymphocytes at 6, 12, and 24 h after treatment.

CAT activities(U/mgprot)				
time	pairing	activities		*P*
6 h	P — M	8.864 ± 0.060	8.132 ± 0.059	<0.001
	M — Se	8.132 ± 0.059	8.212 ± 0.032	0.036
	M — D1	8.132 ± 0.059	5.826 ± 0.029	<0.001
	M — D2	8.132 ± 0.059	6.102 ± 0.029	<0.001
	M — D3	8.132 ± 0.059	6.832 ± 0.033	<0.001
	M — D4	8.132 ± 0.059	8.168 ± 0.111	0.357
	D1 — SD1	5.826 ± 0.029	5.893 ± 0.019	0.007
	D2 — SD2	6.102 ± 0.029	6.522 ± 0.006	0.001
	D3 — SD3	6.832 ± 0.033	6.990 ± 0.013	0.005
	D4 — SD4	8.168 ± 0.111	8.357 ± 0.109	<0.001
12 h	P — M	8.594 ± 0.068	7.725 ± 0.037	<0.001
	M — Se	7.725 ± 0.037	8.079 ± 0.077	0.004
	M — D1	7.725 ± 0.037	4.858 ± 0.044	<0.001
	M — D2	7.725 ± 0.037	5.628 ± 0.034	<0.001
	M — D3	7.725 ± 0.037	6.185 ± 0.006	<0.001
	M — D4	7.725 ± 0.037	7.448 ± 0.028	<0.001
	D1 — SD1	4.858 ± 0.044	5.111 ± 0.020	0.012
	D2 — SD2	5.628 ± 0.034	6.064 ± 0.007	0.001
	D3 — SD3	6.185 ± 0.006	6.567 ± 0.036	0.002
	D4 — SD4	7.448 ± 0.028	8.063 ± 0.059	0.001
24 h	P — M	7.493 ± 0.054	6.206 ± 0.552	0.061
	M — Se	6.206 ± 0.552	6.349 ± 0.057	0.707
	M — D1	6.206 ± 0.552	3.545 ± 0.005	0.014
	M — D2	6.206 ± 0.552	3.901 ± 0.027	0.019
	M — D3	6.206 ± 0.552	4.480 ± 0.029	0.034
	M — D4	6.206 ± 0.552	5.416 ± 0.024	0.138
	D1 — SD1	3.545 ± 0.005	3.817 ± 0.020	0.001
	D2 — SD2	3.901 ± 0.027	4.438 ± 0.047	<0.001
	D3 — SD3	4.480 ± 0.029	5.289 ± 0.019	<0.001
	D4 — SD4	5.416 ± 0.024	5.573 ± 0.036	0.002

**Table 6:** Effects of DON and/or Na_2_SeO_3_ on the levels of GSH in GPX1-knockdown porcine splenic lymphocytes at 6, 12, and 24 h after treatment.

GSH (umol/gprot)				
time	pairing	content		*P*
6 h	P — M	239.414 ± 6.165	233.345 ± 3.805	0.047
	M — Se	233.345 ± 3.805	233.260 ± 4.220	0.757
	M — D1	233.345 ± 3.805	142.407 ± 3.220	<0.001
	M — D2	233.345 ± 3.805	168.664 ± 4.832	<0.001
	M — D3	233.345 ± 3.805	204.520 ± 4.570	<0.001
	M — D4	233.345 ± 3.805	226.293 ± 3.950	<0.001
	D1 — SD1	142.407 ± 3.220	142.290 ± 3.005	0.446
	D2 — SD2	168.664 ± 4.832	168.965 ± 4.895	0.015
	D3 — SD3	204.520 ± 4.570	205.786 ± 4.415	0.005
	D4 — SD4	226.293 ± 3.950	226.867 ± 4.311	0.110
12 h	P — M	229.384 ± 5.665	222.599 ± 4.575	0.008
	M — Se	222.599 ± 4.575	221.932 ± 5.550	0.358
	M — D1	222.599 ± 4.575	126.747 ± 3.780	<0.001
	M — D2	222.599 ± 4.575	153.548 ± 3.495	<0.001
	M — D3	222.599 ± 4.575	179.396 ± 3.815	<0.001
	M — D4	222.599 ± 4.575	198.856 ± 4.415	<0.001
	D1 — SD1	126.747 ± 3.780	127.745 ± 2.195	0.389
	D2 — SD2	153.548 ± 3.495	153.221 ± 1.760	0.772
	D3 — SD3	179.396 ± 3.815	181.746 ± 2.890	0.048
	D4 — SD4	198.856 ± 4.415	202.112 ± 3.990	0.006
24 h	P — M	176.528 ± 2.395	165.320 ± 3.455	0.003
	M — Se	165.320 ± 3.455	169.596 ± 2.640	0.012
	M — D1	165.320 ± 3.455	97.760 ± 1.326	<0.001
	M — D2	165.320 ± 3.455	113.939 ± 2.205	<0.001
	M — D3	165.320 ± 3.455	133.746 ± 3.235	<0.001
	M — D4	165.320 ± 3.455	148.813 ± 3.545	<0.001
	D1 — SD1	97.760 ± 1.326	108.301 ± 2.090	0.002
	D2 — SD2	113.939 ± 2.205	131.858 ± 3.510	0.002
	D3 — SD3	133.746 ± 3.235	147.576 ± 2.850	<0.001
	D4 — SD4	148.813 ± 3.545	156.226 ± 2.585	0.006

**Table 7:** Effects of DON and/or Na_2_SeO_3_ on T-AOC in GPX1-knockdown porcine splenic lymphocytes at 6, 12, and 24 h after treatment.

T-AOC (U/mg.prot)				
time	pairing	numerical		*P*
6 h	P — M	35.412 ± 0.445	32.571 ± 0.464	<0.001
	M — Se	32.571 ± 0.464	32.749 ± 0.422	0.018
	M — D1	32.571 ± 0.464	17.694 ± 0.257	<0.001
	M — D2	32.571 ± 0.464	19.262 ± 0.233	<0.001
	M — D3	32.571 ± 0.464	22.818 ± 0.189	<0.001
	M — D4	32.571 ± 0.464	31.748 ± 0.386	0.003
	D1 — SD1	17.694 ± 0.257	18.089 ± 0.259	<0.001
	D2 — SD2	19.262 ± 0.233	19.759 ± 0.252	<0.001
	D3 — SD3	22.818 ± 0.189	23.566 ± 0.324	0.011
	D4 — SD4	31.748 ± 0.386	32.308 ± 0.343	0.002
12 h	P — M	23.787 ± 0.314	20.108 ± 0.212	<0.001
	M — Se	20.108 ± 0.212	21.821 ± 0.252	<0.001
	M — D1	20.108 ± 0.212	9.731 ± 0.094	<0.001
	M — D2	20.108 ± 0.212	10.618 ± 0.106	<0.001
	M — D3	20.108 ± 0.212	14.833 ± 0.271	<0.001
	M — D4	20.108 ± 0.212	17.613 ± 0.219	<0.001
	D1 — SD1	9.731 ± 0.094	10.002 ± 0.139	0.009
	D2 — SD2	10.618 ± 0.106	11.025 ± 0.111	<0.001
	D3 — SD3	14.833 ± 0.271	15.449 ± 0.170	0.009
	D4 — SD4	17.613 ± 0.219	19.087 ± 0.353	0.003
24 h	P — M	15.915 ± 0.167	13.107 ± 0.169	<0.001
	M — Se	13.107 ± 0.169	15.327 ± 0.184	<0.001
	M — D1	13.107 ± 0.169	5.928 ± 0.064	<0.001
	M — D2	13.107 ± 0.169	6.647 ± 0.079	<0.001
	M — D3	13.107 ± 0.169	7.817 ± 0.089	<0.001
	M — D4	13.107 ± 0.169	9.373 ± 0.095	<0.001
	D1 — SD1	5.928 ± 0.064	6.374 ± 0.077	<0.001
	D2 — SD2	6.647 ± 0.079	7.586 ± 0.090	<0.001
	D3 — SD3	7.817 ± 0.089	9.350 ± 0.167	0.001
	D4 — SD4	9.373 ± 0.095	11.210 ± 0.050	<0.001

**Table 8:** Effects of DON and/or Na_2_SeO_3_ on the cellular capacity to inhibit hydroxyl radicals in GPX1-knockdown porcine splenic lymphocytes at 6, 12, and 24 h after treatment.

The capacity of inhibing effect of hydroxyl radical (U/mg.prot)				
time	pairing	numerical		*P*
6 h	P — M	192.734 ± 6.195	173.766 ± 4.325	0.003
	M — Se	173.766 ± 4.325	177.184 ± 3.675	0.012
	M — D1	173.766 ± 4.325	110.845 ± 3.445	<0.001
	M — D2	173.766 ± 4.325	116.058 ± 2.265	<0.001
	M — D3	173.766 ± 4.325	142.478 ± 3.595	<0.001
	M — D4	173.766 ± 4.325	170.113 ± 4.335	<0.001
	D1 — SD1	110.845 ± 3.445	111.664 ± 3.685	0.027
	D2 — SD2	116.058 ± 2.265	117.824 ± 3.725	0.171
	D3 — SD3	142.478 ± 3.595	146.309 ± 3.480	<0.001
	D4 — SD4	170.113 ± 4.335	171.357 ± 3.805	0.056
12 h	P — M	172.825 ± 3.715	148.956 ± 4.235	<0.001
	M — Se	148.956 ± 4.235	156.173 ± 1.685	0.039
	M — D1	148.956 ± 4.235	73.068 ± 1.841	<0.001
	M — D2	148.956 ± 4.235	89.649 ± 1.747	0.001
	M — D3	148.956 ± 4.235	118.062 ± 1.585	0.002
	M — D4	148.956 ± 4.235	143.714 ± 3.145	0.014
	D1 — SD1	73.068 ± 1.841	74.280 ± 2.008	0.006
	D2 — SD2	89.649 ± 1.747	91.678 ± 2.932	0.097
	D3 — SD3	118.062 ± 1.585	122.214 ± 2.665	0.022
	D4 — SD4	143.714 ± 3.145	145.834 ± 2.895	0.005
24 h	P — M	154.714 ± 3.225	130.376 ± 2.065	0.001
	M — Se	130.376 ± 2.065	140.390 ± 2.380	<0.001
	M — D1	130.376 ± 2.065	53.646 ± 2.155	<0.001
	M — D2	130.376 ± 2.065	72.114 ± 2.847	<0.001
	M — D3	130.376 ± 2.065	94.263 ± 1.722	<0.001
	M — D4	130.376 ± 2.065	120.947 ± 2.861	0.002
	D1 — SD1	53.646 ± 2.155	55.415 ± 1.853	0.010
	D2 — SD2	72.114 ± 2.847	77.168 ± 1.659	0.018
	D3 — SD3	94.263 ± 1.722	102.263 ± 2.389	0.002
	D4 — SD4	120.947 ± 2.861	125.143 ± 2.625	0.001

The rates of change in SOD, CAT, GSH, H_2_O_2_, MDA, T-AOC, and the inhibition of free hydroxyl radicals are shown in Tables 9–15. Except in a few cases, most KG-An(the rates of change in the GPX1-knockdown porcine splenic lymphocytes with knockdown group An) are less than NG-An(the change rates of our early achievements were normal group An). The levels of ROS are shown in Table 16. The level of ROS was lowest in group P, whereas group D1 had the highest ROS content. The ROS content was significantly higher in group M than in group P. The ROS content was significantly higher in groups D1–4 than in group M, except for group D4. The ROS content was significantly lower in group Se than that in group M. When the cells were treated with both DON and Na_2_SeO_3_, the ROS content was significantly lower in the groups SD1–4 than in the groups D1–4, except for group SD1.

**Table 9:** Rates of change in the H_2_O_2_ contents of GPX1-knockdown porcine splenic lymphocytes and normal porcine splenic lymphocytes at 6, 12, and 24 h after treatment.

Groups	The change rates of H_2_O_2_(%)		
	6 h	12 h	24 h
KG-A1	−1.917	−2.598	−5.176
NG-A1	−2.852	−3.877	−5.525
KG-A2	−3.289	−6.008	−8.123
NG-A2	−5.263	−7.281	−5.863
KG-A3	−4.968	−6.737	−14.443
NG-A3	−6.245	−8.660	−14.864
KG-A4	−12.160	−4.397	−19.517
NG-A4	−2.354	−5.215	−19.201

**Table 10:** Rates of change in the MDA content of GPX1-knockdown porcine splenic lymphocytes and normal porcine splenic lymphocytes at 6, 12, and 24 h after treatment.

Groups	The change rates of MDA(%)		
	6 h	12 h	24 h
KG-A1	−1.207	−1.946	−3.395
NG-A1	−2.442	−2.659	−5.212
KG-A2	−2.565	−2.760	−8.065
NG-A2	−3.550	−3.751	−9.448
KG-A3	−3.241	−3.785	−6.212
NG-A3	−4.470	−4.977	−9.614
KG-A4	−1.857	−4.882	−7.460
NG-A4	−2.937	−5.637	−10.618

**Table 11:** Rates of change in the SOD activity of GPX1-knockdown porcine splenic lymphocytes and normal porcine splenic lymphocytes at 6, 12, and 24 h after treatment.

Groups	The change rates of SOD(%)		
	6 h	12 h	24 h
KG-A1	0.504	1.241	3.726
NG-A1	0.620	1.549	4.307
KG-A2	1.923	2.279	4.263
NG-A2	2.106	2.419	5.496
KG-A3	2.614	2.848	19.475
NG-A3	2.980	3.065	24.023
KG-A4	3.231	6.887	17.331
NG-A4	3.086	4.978	25.139

**Table 12:** Rates of change in the CAT activity in GPX1-knockdown porcine splenic lymphocytes and normal porcine splenic lymphocytes at 6, 12, and 24 h after treatment.

Groups	The change rates of CAT(%)		
	6 h	12 h	24 h
KG-A1	1.150	5.208	7.673
NG-A1	5.345	5.780	9.308
KG-A2	6.867	7.676	13.740
NG-A2	8.320	9.964	18.559
KG-A3	2.313	6.176	18.058
NG-A3	10.657	12.255	19.103
KG-A4	2.301	8.257	2.899
NG-A4	2.222	10.774	12.278

**Table 13:** Rates of change in the GSH content of GPX1-knockdown porcine splenic lymphocytes and normal porcine splenic lymphocytes at 6, 12, and 24 h after treatment.

Groups	The change rates of GSH(%)		
	6 h	12 h	24 h
KG-A1	0.082	0.079	10.783
NG-A1	0.982	0.988	18.039
KG-A2	0.179	−0.213	15.727
NG-A2	1.615	1.598	18.957
KG-A3	0.619	1.310	29.742
NG-A3	1.883	1.861	10.293
KG-A4	0.254	1.637	4.981
NG-A4	2.013	2.078	8.850

**Table 14:** Rates of change in the T-AOC of GPX1-knockdown porcine splenic lymphocytes and normal porcine splenic lymphocytes at 6, 12, and 24 h after treatment.

Groups	The change rates of T-AOC(%)		
	6 h	12 h	24 h
KG-A1	2.226	2.788	7.521
NG-A1	2.493	3.161	9.560
KG-A2	2.575	3.837	14.126
NG-A2	2.999	4.893	17.197
KG-A3	3.276	4.155	19.611
NG-A3	3.531	5.866	26.060
KG-A4	1.176	8.369	19.599
NG-A4	1.391	9.268	27.709

**Table 15:** Rates of change in the capacities of GPX1-knockdown porcine splenic lymphocytes and normal porcine splenic lymphocytes to inhibit hydroxyl radicals at 6, 12, and 24 h after treatment.

Groups	The change rates of inhibition of hydroxyl radical (%)		
	6 h	12 h	24 h
KG-A1	0.739	1.659	3.288
NG-A1	1.189	2.261	5.863
KG-A2	1.522	2.263	7.007
NG-A2	2.396	4.188	9.276
KG-A3	2.689	3.517	8.486
NG-A3	4.788	5.704	10.520
KG-A4	0.731	1.475	3.469
NG-A4	1.033	3.149	5.068

**Table 16:** Effects of DON and/or Na_2_SeO_3_ on the levels of ROS in GPX1-knockdown porcine splenic lymphocytes at 24 h after treatment.

ROS content				
Time	pairing	median		*P*
24 h	P — M	847.00 ± 42.00	1069.00 ± 52.00	0.001
	M — Se	1069.00 ± 52.00	893.33 ± 22.50	0.009
	M — D1	1069.00 ± 52.00	1426.33 ± 37.50	0.001
	M — D2	1069.00 ± 52.00	1320.33 ± 41.50	0.001
	M — D3	1069.00 ± 52.00	1241.33 ± 61.50	0.001
	M — D4	1069.00 ± 52.00	1060.00 ± 67.00	0.408
	D1 — SD1	1426.33 ± 37.50	1390.00 ± 27.00	0.027
	D2 — SD2	1320.33 ± 41.50	1271.33 ± 47.50	0.005
	D3 — SD3	1241.33 ± 61.50	1083.66 ± 95.00	0.015
	D4 — SD4	1060.00 ± 67.00	1078.00 ± 45.00	0.276

## Discussion

Oxidative stress occurs when the concentration of ROS exceeds the antioxidant capacity of the cell. When cells cultured *in vitro* are subjected to oxidative stress, they are mainly protected by the enzymes of their own antioxidant system, predominantly SOD, CAT, and GPX. GPX1 is the main GPX in spleen lymphocytes, and plays an important role in protecting the cells against oxidative stress. Using GSH as its substrate, GPX1 participates in the reduction of toxic peroxides, promotes the decomposition of H_2_O_2_, and thus protects the cell membrane. Yan^25^ knocked down the expression of GPX1 in ATDC5 cells with small hairpin RNA (shRNA), and found that the antioxidant capacity of the cells decreased. Our results are similar insofar as after GPX1 expression was reduced, the H_2_O_2_ content in group M increased as the incubation time increased, relative to that in group P, even at the beginning of silence that the SOD and CAT might compensate. The MDA and ROS content of group M was significantly higher than that of group P throughout the whole experiment (*P* < 0.01), whereas the GSH, SOD and CAT activities, T-AOC, and the capacity of the cells to inhibit hydroxyl radicals were significantly lower in group M. After GPX1 expression was knocked down in the porcine splenic lymphocytes, the antioxidant capacity of the cells decreased compared with that in group P, and the oxidative stress in the cells caused them more damage.

The presence of large amounts of lipids in cells makes them highly susceptible to peroxide and the damage caused by oxidative stress. The many lipid peroxidation products generated also have a toxic effect on the cells, causing further damage. The cellular levels of important lipid peroxidation products, including MDA, indicate the degree of lipid peroxidation and the amounts of free oxygen radicals in the cells, and can be used to indirectly determine the degree of oxidative damage to them^26^. When Kouadio *et al*.^27^ added 5–40 μM DON to the Caco-2 cell line, there was a significant increase in the MDA content after 24 h. Li^28^ also showed a significant increase in the MDA content after adding 100–2000 ng/mL DON to a chicken embryo fibroblasts (DF-1 cells) for 6–48 h. In the present study, the content of MDA in the GPX1-knockdown porcine splenic lymphocytes increased as the DON concentration and the culture period increased. Therefore, our results are similar to those of the studies described above. We compared the results of this experiment with the results of our experiment with prophase cells^19^. After treatment with DON, the MDA content in the GPX1-knockdown porcine splenic lymphocytes was significantly higher than in the normal porcine splenic lymphocytes, indicating that lipid peroxidation increased in the cells after GPX1 knockdown. After the addition of DON, the content of H_2_O_2_ was significantly higher in the GPX1-knockdown porcine splenic lymphocytes than in the normal porcine splenic lymphocytes because GPX1 decomposes H_2_O_2_ and thus reduces DON-induced oxidative stress. T-AOC reflects the overall antioxidant capacity of the cells and is a comprehensive indicator of the cells antioxidant system. In this study, the T-AOC of the GPX1-knockdown porcine splenic lymphocytes decreased as the DON concentration and the incubation time increased, and was significantly lower than that in the normal porcine splenic lymphocytes treated with the same concentrations of DON for the same culture periods (*P* < 0.01). The results of this study are similar to those of Hao *et al*.^29^. who added AFB1 to lymphocytes from the spleens of pigs in which GPX1 was knocked down. Therefore, DON causes the levels of MDA and H_2_O_2_ to increase and the cellular T-AOC to decrease more severely in GPX1-knockdown porcine splenic lymphocytes than in control cells. Our results also show that, compared with the normal porcine splenic lymphocytes, the capacity of the GPX1-knockdown cells to inhibit hydroxyl radicals decreased more dramatically as the DON concentration increased and the incubation time increased, resulting in a greater accumulation of free radicals, a greater degree of oxidative stress, a greater reduction in T-AOC, and therefore more-severe oxidative damage.

Oxidative damage occurs when the intracellular reactive oxygen concentration exceeds the cell’s antioxidant capacity. ROS mainly include superoxide anions (∙O_2_^−^), H_2_O_2_, and the hydroxyl radical (−OH). Cells scavenge ROS through both enzymatic and non-enzymatic pathways. The enzymatic pathways consist of antioxidant enzymes such as SOD, CAT, and GPX, and the non-enzymatic pathways involve GSH, Se, vitamin C, vitamin E, and β-carotene^30^. SOD uses the superoxide anion (∙O_2_^−^) produced in cells as its substrate, producing reduced SOD (SOD^−^) and O_2_, and then SOD^−^ reacts with ∙O_2_^−^ to produce SOD and H_2_O_2_. H_2_O_2_ is then catalysed by CAT and GPX to generate H_2_O and O_2_^31^, thus protecting the cell membrane from damage. CAT is a terminal oxidase that catalyses the decomposition of H_2_O_2_ into H_2_O and O_2_. GSH is a co-substrate of GPX, which catalyses it to GSSG, thus reducing a toxic peroxide to a nontoxic hydroxyl compounds, and at the same time promoting the decomposition of H_2_O_2_. This protects the cell membrane structure and function are safe from the oxide interference and damage. Studies have shown that at lower GSH contents can result in decreased GPx1 activity^32^. Therefore, after reactive oxygen is produced in cells, SOD acts as the first line of defence and CAT and GPX as the second line of defence, acting together in the process of scavenging intracellular reactive oxygen. Our results show that DON caused the activities of SOD and CAT to increase, and reduced the levels of GSH as the DON concentration and incubation time increased, demonstrating the time and concentration dependence of its effects. The results of this study are similar to those of Gan *et al*.^33^, who showed that when the expression of the GPX1 protein was knocked down, the GSH content of the cells decreased significantly after ochratoxins (OTA) were added. When the results of the present study were compared with the results of our study of prophase cells^19^, the SOD and CAT activities and the levels of GSH were significantly lower in the GPX1-knockdown porcine splenic lymphocytes when same concentrations of DON were added and the cells were cultured for the same time. This may be because the cells themselves had a lower antioxidant capacity after GPX1 knockdown, and the intracellular accumulation of ROS and the consumption of antioxidant enzymes and GSH were increased by the cytotoxicity of DON and DON-induced oxidative stress.

Selenium is a necessary trace element in the diet of mammals because it plays an important role in many organ systems and life activities. The antioxidant effects of Se have always been a research hotspot, and it mainly occurs in selenocysteine and selenomethionine in selenoproteins, where it plays its antioxidant role. GPX is the main selenium antioxidant enzyme in cells^34^. GPX has at least four isoenzymes, and GPX1 is the most strongly expressed GPX in porcine splenic lymphocytes, where it plays an important role in ameliorating oxidative stress. In this study, after knocking down GPX1 expression, we added Na_2_SeO_3_ to the group M cells, and showed that the levels of MDA and H_2_O_2_ are significantly lower, and the activities of SOD and CAT, the levels of GSH and T-AOC, and the capacity to inhibit hydroxyl radicals were significantly higher than group M. These results are similar to the results of Tang^35^, who showed that Na_2_SeO_3_ had an antagonistic effect on GPX1-knockdown-induced oxidative damage to porcine splenic lymphocytes.

A large number of studies have shown that Se prevents the oxidative stress induced by some mycotoxins^36–38^. In this study, the levels of MDA and H_2_O_2_ were significantly lower in the groups SD1–4 than in the groups D1–4 (*P* < 0.05 or *P* < 0.01), and the activities of SOD and CAT, the levels of GSH, T-AOC, and the capacity of the cells to inhibit hydroxyl radicals were higher in the groups SD1–4 than in the groups D1–4 mostly (*P* < 0.05 or *P* < 0.01). The rates of these changes in GPX1 knockdown porcine splenic lymphocytes were greater than in the normal porcine splenic lymphocytes, note the elevated ratio of the activities of SOD and CAT, the levels of GSH, T-AOC and the capacity of cells to inhibit hydroxyl radicals, the reduced ratio of the levels of MDA and H_2_O_2_ of GPx1 knockdown porcine splenic lymphocytes are lower than porcine splenic lymphocytes. These data indicate that the protective effects of Na_2_SeO_3_ against DON-induced oxidative damage were reduced by GPX1 knockdown.

In summary, our results demonstrate that the knockdown the GPX1 in porcine splenic lymphocytes reduces their anti-oxidative capacity, and the cells’ own oxidative stress causes them more damage than is caused in normal cells’. DON caused greater oxidative damage in GPX1-knockdown porcine splenic lymphocytes than in normal control cells. When combined with DON, Na_2_SeO_3_ ameliorated the DON-induced oxidative damage to GPX1-knockdown porcine splenic lymphocytes, but its protective effects were less marked than in normal cells. In the future, we will overexpress the GPX1 gene to in-depth study its effects, or to study spleen lymphocyte organelles. These studies are required to understand the molecular mechanisms underlying these phenomena. Our results also suggest that improved nutrition may be a novel approach to mitigating mycotoxin contamination in animal production.

## Materials and Methods

### Reagents

Foetal bovine serum was purchased from Gibco/Life Technologies (California, USA). Na_2_SeO_3_ powder was purchased from Xiya Reagent (Chengdu, China). DON was obtained from Sigma-Aldrich (USA). RPMI-1640 medium was obtained from Boster Biological Technology Co., Ltd (Wuhan, China). Hank’s solution and lymphocyte separation medium were obtained from Tianjin Hao Yang Biological Institute (China). Kits for testing glutathione(GSH), malonaldehyde (MDA), total antioxidant capacity (T-AOC), glutathione peroxidase (GPx), superoxide dismutase (SOD), catalase (CAT), hydrogen peroxide (H_2_O_2_), Hydroxyl Free Radical, were obtained from the Nanjing Jiancheng Bioengineering Institute (Nanjing, China). The Reactive Oxygen Species Assay Kit was obtained from Beyotime Biotechnology (Shanghai, China). TRIzol Reagent was purchased from Invitrogen Biotechnology Co., Ltd (Shanghai, China). PrimeScript™ RT Reagent Kit and SYBR® Premix Ex Taq™ II were purchased from Takara (Shiga, Japan). The anti-GPX1 primary antibody (ab50427) and the rabbit anti-goat IgG H& L secondary antibody (ab6741) were from abcam.

### Production and treatment of porcine splenic lymphocytes and the establishment of GPX1-knockdown porcine spleen lymphocytes

For a description of the production of the porcine spleen lymphocytes, refer to our earlier paper^24^. All study procedures were approved by the Institutional Animal Care and Use Committee of Sichuan Agricultural University. All experiments were performed in accordance with relevant guidelines and regulations. Based on a published sequence of porcine GPX1 mRNA (GenBank NM-214201.0), siRNA was designed using Block-iT^TM^ siRNA RNAi Designer (Thermo Fisher Scientific). The sequence with the highest score was selected, which had the control siRNA sequence 5′-GGGACUACACCCAGAUGAATT-3′. The scrambled siRNA was synthesized by Thermo Fisher Scientific, with the sequence 5′-UUCGUAUCUGGGUGUACCCTT-3′. The control siRNA sequence was confirmed to be consistent with that reported by Gan *et al*.^33^. The FAM fluorescent marker was added to the siRNA as required. RFect^PM^ small nucleotide transfection agents was used for the transfection. To determine the optimal concentration of siRNA and transfection reagent, we tested four combinations, according to the reagent instructions. The specific information is shown in Table 17. The cells with the highest transfection efficiency were used for the subsequent experiments. The expression of GPX1 mRNA was detected with quantitative real-time PCR (qPCR) and the expression of GPX1 protein was detected 48 h after transfection with western blotting.

**Table 17:** Concentrations of transfection reagent and siRNA.

Combination	names	①	②	③	④
reagent		10 μL	12 μL	14 μL	16 μL
Positive siRNA(with FAM)	GPx1	12 nmol/L	18 nmol/L	24 nmol/L	30 nmol/L

The prepared porcine spleen lymphocytes and GPX1-knockdown lymphocytes were cultured in triplicate in six-well tissue culture plates at 3 × 10^6^ cells/mL. To determine the effects of DON and Se^14,24^, 11 groups of both types of cells were treated with medium only or with DON and/or Se in the following combinations: Group P (porcine splenic lymphocytes), group M (GPX1-knockdown porcine splenic lymphocytes), D1 (824 ng/mL DON), D2 (412 ng/mL DON), D3 (206 ng/mL DON), D4 (103 ng/mL DON), Se (2 μmol/L Na_2_SeO_3_), SD1 (2 μmol/L Na_2_SeO_3_ + 824 ng/mL DON), SD2 (2 μmol/L Na_2_SeO_3_ + 412 ng/mL DON), SD3 (2 μmol/L Na_2_SeO_3_ + 206 ng/mL DON), and SD4 (2 μmol/L Na_2_SeO_3_ + 103 ng/mL DON). The cells were incubated for 6, 12, or 24 h. The concentration of DON and Se and the time of cells were incubated have been determined in the early stage of the laboratory. And the antioxidant indices were determined at each time point. The levels of ROS were detected at 24 h.

### Flow-cytometric determination of positive siRNA transfection efficiency

After transfection for 24 h, the cells were collected with centrifugation at 1800 r/min for 5 min at 4 °C. The supernatant, was discarded and the cells were washed twice with phosphate-buffered saline (PBS) at 4 °C. The PBS cell suspension (100 μL) was precooled to 4 °C and filtered through a 300 mesh filter. The cells were then analysed with flow cytometry.

### Reverse transcription (RT)–qPCR analysis of GPX1 mRNA expression after siRNA transfection

For a description of the RT–qPCR performed, see the paper by Wang^26^. The primer sequencing for GPx1: (F- TGGGGAGATCCTGAATTG, R- GATAAACTTGGGGTCGGT) β-Actin was used as reference gence: (F- CTGCGGCATCCACGAAACT, R- AGGGCCGTGATCTCCTTCTG).

### Detection of GPX1 protein with western blotting after transfection

The cells were washed twice with precooled PBS and suspended in 300 μL of PBS. The cells were collected and homogenized on ice, and phenylmethanesulfonyl fluoride was added to the protein lysates. After 40 min on ice, the lysates were centrifuged at 12,000 rpm for 40 min at 4 °C and the supernatants collected. The cellular protein was quantified with the BCA method using bovine serum albumin as the standard. Samples of protein (50 μg) were diluted in sample loading buffer and heated at 95 °C for 5 min. The denatured proteins were separated with 10% sodium dodecyl sulfate-polyacrylamide gel electrophoresis (SDS-PAGE), transferred onto polyvinylidene difluoride membrane, and placed in closed liquid for 1 h at 37 °C. The primary antibody was added and the membranes incubated at 4 °C. At the same time, another membrane was incubated without antibody in Tris-buffered saline containing Tween 20 (TBS-T) as the negative control. After repeated washes, the membrane was incubated with a rabbit anti-goat IgG H&L secondary antibody with gentle shaking for 1 h at room temperature. After the membrane was washed, used western blot mark to observe, the absorbance (A) values were quantitatively analysed with an image analysis.

### Determination of antioxidant indices and levels of ROS

The antioxidant indices and the levels of ROS in the cell preparations (supernatants, cell lysates, and cells) were measured according to the protocols of the corresponding kits.

### Statistical analysis

The test results are expressed as means standard ± deviations. Excel was used to preliminarily test and collate the results. The statistical software SPSS ver. 22 was used for later statistical analyses, and Duncan’s method was used for multiple comparisons. The rates of change in some antioxidant indices, including H_2_O_2_, MDA, SOD, CAT, GSH, T-AOC, and the inhibition of hydroxyl radicals, were calculated in the SD and Group D1–4s (as follows). To express the rates of change in the GPX1-knockdown porcine splenic lymphocytes with knockdown group An(KG-An), the change rates of our early achievements were normal group An(NG-An). The changes in the antioxidant indices after Na_2_SeO_3_ was added were calculated by comparing the absolute values of KG-An with NG-An, to determine whether Na_2_SeO_3_ was antagonistic to the porcine spleen lymphocyte by GPX1.

$${\rm{An}}=({\rm{SD}}1-4/D1-4-1)\times 100 \% \,({\rm{n}}=4)$$

## Electronic supplementary material

Dataset

## Data Availability

All data generated or analysed are valid during this study, included in this published article (and its Supplementary Information files).
